# Advanced 2D Materials for Emerging Applications

**DOI:** 10.3390/nano15231823

**Published:** 2025-12-02

**Authors:** Maiyong Zhu

**Affiliations:** School of Materials Science & Engineering, Jiangsu University, Zhenjiang 212013, China; maiyongzhu@ujs.edu.cn

Since the discovery of graphene in 2004, two-dimensional (2D) materials have received increasing attention owing to their unique electronic, optical, mechanical, and chemical properties. To date, the family of 2D materials has been greatly enlarged by developing numerous emerging members, including graphdiyne, transition metal dichalcogenides (TMDs), graphitic carbon nitride (g-C_3_N_4_), hexagonal boron nitride (h-BN), black phosphorus (BP), MXenes, metallene, etc.

The Special Issue “Advanced 2D materials for emerging applications” represents a significant contribution to the field of 2D materials, showcasing 10 innovative studies covering a broad description of the various synthetic approaches and emerging applications. This collection offers a multidisciplinary perspective on the future of 2D materials.

The motivation behind this Special Issue is rooted in constructing various devices that take advantage of 2D materials which possess tunable interlayer spacings, adjustable terminal surface groups, and abundant surface areas to accommodate guest species, all while environmentally sustainable. The manuscripts featured in this Special Issue cover the general principles and applications of 2D materials in a wide range of devices, including supercapacitors, reconfigurable electronics, zinc-ion batteries, and radiofrequency devices. The materials discussed in the collection include graphene, TMS, MXene, and high-entropy alloys. Additionally, some papers focus on advanced manufacturing processes and strategies to improve the performance of 2D materials, such as constructing heterojunctions and introducing vacancies. Each contribution emphasizes the significance of 2D materials in boosting long-term stability, biocompatibility, and the need to be environmentally friendly.

We will now summarize these contributions in this editorial of this Special Issue. Pan et al. [1] provide a comprehensive review, discussing the applications of 2D-material-based reconfigurable electronics in logic operation and artificial intelligence summarizations by focusing on the working principles of 2D material devices used for reconfigurable electronics. Rahimi et al. [2] developed an automated pipeline for the prediction of antibacterial potential based on graphene–polymer composites. Hwang et al. [3] reported a radio frequency antenna device using single-layer graphene, which is capable of changing resonant frequency. Yang et al. [4] developed a hybrid consisting of meso-Cu-MOF and GO-COOH, in which GO-COOH serves as a substrate to support meso-Cu-MOF. When evaluated as a supercapacitor electrode, the resulting meso-Cu-MOF@GO-COOH hybrid delivers a higher capacitance of 292.5 F g^−1^. Moreover, an asymmetric supercapacitor device is further assembled in order to verify the potential for practical application, which offers a capacitance of 63 F g^−1^ (0.5 A g^−1^), an energy density of 27.7 Wh kg^−1^, and a power density of 496.8 W kg^−1^. Furthermore, the same group [5] also extended the principle to Ti_3_C_2_T_x_, another important 2D material. In order to improve the structural stability of the hybrid, 2,6-diaminopyridine (DAP) and urea pyrimidinone isocyanate (UPy-NCO) units are introduced to modify the structure and properties. An electrochemical investigation indicates that the Cu-MOF@Ti_3_C_2_T_X_-20%DAP-UPy hybrid exhibited excellent performance in terms of specific capacitance (148 F g^−1^ at 1 A g^−1^), capacitance retention (88% as the current density increased from 0.2 to 5 A g^−1^), and cycling stability (91.1% after 5000 cycles at 1 A g^−1^).

Another important 2D material discussed in this Special Issue is metallic sulfide. Currently, much attention is focused on aqueous zinc-ion batteries due to the safety issues of lithium-ion batteries. Xu et al. [6] developed an oxygen-assisted method to synthesize phosphorus (P)-atom-embedded, three-dimensional marigold-shaped 1T MoS_2_ structures (P-MoS_2_), demonstrating excellent performance in a rechargeable zinc-ionic battery. The resulting P-MoS_2_ possesses S vacancies (Sv). Owing to the embedment of P, the interlayer spacing of P-MoS_2_ is expanded, leading to the strengthening of Zn^2+^ intercalation/deintercalation. Furthermore, the three-dimensional marigold-shaped structure with 1T phase retains an internal free space, can adapt to the volume change during charge and discharge, and improves the overall conductivity. Owing to the unique layer structures, 2D materials show great potential in the fabrication of van der Waals (vdW) heterojunctions, leading to many fascinating properties. Li et al. [7] investigated group-III selenide van der Waals (vdW) heterojunctions consisting of 2D α-In_2_Se_3_ and α-Ga_2_Se_3_ ferroelectric (FE) semiconductors employing first-principles calculations, including structural stability, electrostatic potential, interfacial charge transfer, and electronic band structures. Li et al. [8] fabricated a saturable absorber (SA) for a bulk Er:SrF_2_ laser based on a few-layer SnS_2_. When the average output power was 140 mW, the passively Q-switched laser achieved the shortest pulse width at 480 ns, an optimal single pulse energy at 3.78 µJ, and the highest peak power at 7.88 W. The results of the passively Q-switched laser revealed that few-layer SnS_2_ had an admirable non-linear optical response, nearing a 3 μm mid-infrared solid-state laser.

For any application, the fabrication of materials is critical. For 2D materials, the fabrication at a wafer-level scale is always sought after, which allows reliable and reproducible fabrication of a large volume of devices with predictable properties. Taking this point in mind, Silva et al. [9] presented the fabrication steps for a process that allows the on-wafer fabrication of active and passive radiofrequency (RF) devices enabled by graphene. In their work, two fabrication processes are involved. In the first one, graphene is transferred to a back gate surface using critical point drying to prevent cracks in the graphene. In the second process, graphene is transferred to a flat surface planarized by ion milling, with the gate being buried beneath the graphene. In recent years, high-entropy alloys have attained much attention due to their unique properties. Traditional synthesis processes suffer from several drawbacks. Firstly, samples must be recast and pressed several times under vacuum to achieve homogeneity. Secondly, even after these processes, the elements do not mix with complete miscibility and form amalgamations in certain microscopic regions. In order to improve the synthesis efficiency and the quality of high-entropy alloys, Usman et al. [10] developed a high-vacuum radiofrequency magnetron (HVRF) sputtering process to synthesize a Co_30_Cr_20_Ni_20_Mo_20_Ti_10_ high-entropy alloy.

This Special Issue is intended to serve as a valuable resource for a wide audience of researchers, engineers, industry professionals, and students who are engaged in 2D materials. I am confident that the readers will enjoy these contributions and may be able to find inspiration for their own research within this Special Issue. This series of manuscripts will provide maximum impact and will allow researchers in other areas to apply the same methodologies in order to understand the mechanisms of self-assembly in their systems.

I am grateful to all the authors for submitting their studies to the present Special Issue and for its successful completion. I also thank the *Nanomaterials* reviewers for enhancing the quality and impact of all submitted papers. Finally, I sincerely thank the editorial staff of *Nanomaterials* for their support during the development and publication of this Special Issue.
